# Tissue engineering of the urethra: where are we in 2019?

**DOI:** 10.1007/s00345-019-02826-3

**Published:** 2019-06-12

**Authors:** Christopher Chapple

**Affiliations:** Sheffield Teaching Hospitals, Sheffield, UK

**Keywords:** Tissue engineering, Oral mucosa, Urethroplasty, Urethral stricture

## Abstract

**Purpose:**

The purpose of this review is to assess the potential role of tissue engineering for urethral reconstruction. It is well- recognised that urethrotomy remains the first-line therapy in the treatment of urethral stricture. Following on from the randomised study which recommended no difference between urethrotomy and urethral dilation, Steenkamp et al. reported long-term success rates of only 20%. Patients with longer strictures, penile or distal urethral strictures, and extensive periurethral spongiofibrosis typically do not respond well to repeated incisions. This report reviews the potential role of tissue engineering as applied to augmentation urethroplasty, which is the treatment of choice following failed urethrotomy.

**Methods:**

A review of the literature was carried out. The principal emphasis was on tissue engineering as applied to augmentation urethroplasty, but an introductory section reviews the use of urethrotomy and the background to contemporary practise with augmentation urethroplasty using oral mucosa.

**Results:**

It is evident that a cellular matrix which requires the ingrowth of cells is unlikely to be successful except for very short strictures. Other approaches such as injection of stem cells have not been adequately trialled in humans to date. Tissue-engineered substitute for autologous oral mucosa has been used and the results relating to this are reviewed.

**Conclusions:**

Tissue engineering of autologous tissue for urethroplasty is expensive. It is unnecessary for the majority of cases, but could be potentially useful for very lengthy strictures, for instance, relating to lichen sclerosis. Whilst tissue-engineered oral mucosa has been successfully used, a great deal more work would be necessary to develop an appropriate matrix. Another study has looked at a larger series using an alternative tissue-engineered substitute, but the results have been very disappointing. At present, it has to be concluded that there is no effective and validated tissue engineering solution for the management of urethral stricture disease.

## Introduction

Urethrotomy remains the first-line therapy in the treatment of a urethral stricture. Following on from their randomised study which demonstrated no difference between urethrotomy and urethral dilatation, Steenkamp et al. [1, 2] reported long-term success rates of only 20%. Patients with longer (> 2 cm in length), multiple, penile or distal strictures and extensive periurethral spongio-fibrosis typically did not respond well to repeated incisions. Thus, repeat internal urethrotomy offers no real chance of a cure after a third incision or if the stricture recurs within 3 months of the first incision. Such patients should be offered a urethroplasty—if a short stricture (< 2 cm) an anastomotic procedure which has the highest chance of success, but failing this an augmentation procedure [3]. The intention of this paper is to review the potential for the use of tissue engineering in the management of urethral stricture disease. To do this, the surgical approaches are reviewed followed by an overview of the current evidence related to tissue engineering of the urethra in patients. The potential benefit of tissue engineering over the harvesting of autologous tissue is not needing to harvest the tissue, particularly when long lengths of urethra need to be augmented.

## Beyond urethrotomy

Clearly the best form of tissue engineering would be to augment the healing process associated with an optical urethrotomy. A number of studies have evaluated the efficacy of agents injected into the scar tissue at the site of stricture area as part of an internal urethrotomy procedure to try and reduce recurrence rates by preventing recurrent spongio-fibrosis. Such agents include mitomycin C which has been used for anterior urethral stricture [4]. Authors have reported that after 15 months mean follow-up urethral stricture recurred in 10% of patients in the mitomycin C treated group and in 50% of patients in the untreated group [5]. Another study evaluated the use of triamcinolone injection and showed a significant decrease in recurrence rate [6, 7]. A potential approach is to obtain autologous urothelial or other mature stem cells and use these to augment an urethrotomy. Systematic review of the world literature on this subject concluded that there are a number of regulatory and other technical hurdles which have yet to be overcome before considering this in clinical practice [8–11].

Clearly if an anastomotic urethroplasty is not possible then an augmentation procedure becomes necessary. Currently, grafts are favoured over flaps, sice; although outcomes are similar, flaps are associated with a far greater likelihood of graft site morbidity. A range of materials have been used for urethral grafting including originally penile and scrotal skin which were historically extensively used along with non-genital skin. All of these materials, in particular scrotal skin do carry a high restenosis rate as all are skin types which do not respond well to a wet environment. Additionally, scrotal skin suffers the disadvantage of containing hair follicles which are difficult to successfully remove, leading to the formation of hair in the neo-urethra. Because of these disadvantages the use of ‘wet’ mucosa has been explored leading to the use of oral mucosa, bladder mucosa, and colonic mucosa. Among the tissues successfully used for augmentation urethroplasty, oral mucosa has increasingly gained popularity over the last decade, as it is easy to harvest with low morbidity and does not necessitate invasive surgery. Whilst Humby is often credited with being the first surgeon to describe an oral mucosal graft to augment a strictured urethra in 1941 [12], it is now clear that its first use was described at the end of the ninetieth century by the Russian surgeon Kirill Sapezhko, who reported a series of four cases [13]. Oral mucosa is particularly useful as it represents a ‘wet’ mucosal surface which adapts well to the moist environment of the urethra and is particularly resistant to balanitis xerotica obliterans (BXO). The most equivalent material which has been described for urethroplasty is bladder mucosa, but this requires an invasive approach for its harvesting. It is now widely accepted that the long-term results obtained with the use of penile skin are inferior to oral mucosa and its use is contraindicated in any patients with BXO. Nobody would now dispute the view that scrotal skin is definitely best avoided as the long-term results seen with this ‘dry’ skin are far inferior, because of its propensity to hair bearing and the inevitable transplantation of hair follicles into any reconstruction. The only other major debate in this field of reconstruction relates to whether grafts or flaps of tissue provide superior results. Whilst the results are comparable there is more morbidity seen with flaps [14].

## Tissue engineering

Tissue engineering could be an effective way of facilitating reconstruction without the need for harvesting tissue by generating a bioengineered solution. The options include the use of acellular matrices, seeding cells into a damaged urethra or the implantation of a newly engineered substitute.

Acellular scaffolds have been reported in preclinical studies and certainly enjoyed a vogue. The major problem with this approach is that it is dependent upon the ingrowth of cells. The first material that was reported in preclinical trials was small intestine submucosa (SIS). However, not surprisingly SIS has limited effectiveness when used without stem cell seeding [15, 16]. An organ-specific acellular matrix is another graft option for urethral reconstruction. Urethral acellular matrix (UAM) is collected from the donor, decellularised and the recipient’s urethra is substituted by transplantation. Good results were observed when homologous transplantation was performed. Complete epithelialisation was achieved, and smooth muscle bundles regenerated spontaneously [17]. UAM has been used in clinical experiments with a success rate between 75 and 100% [18]. Another approach was the use of acellular bladder matrix (ABM) harvested from porcine or leporine bladder and seeded with autologous oral keratinocytes, adipose-derived stem cells, autologous bladder smooth muscle cells and urothelial cells [19, 20] and likewise, acellular bladder submucosa matrix (BSM) seeded with autologous minced urethral tissue linked to BSM with fibrin glue [21]. More recently, the use of urine-derived stem cells in a rabbit model has been reported with the potential for this being of value for future research in this area [22]. Translation of these techniques from basic experiments to clinical experience poses numerous challenges for the future. Preclinical data are based on different animal models (rabbit, rat and dog) and various types of injury, none of which accurately mimic the pathological situation seen with a true stricture where there is a full thickness ischaemic spongio-fibrosis. Whilst there have been limited clinical trials, none of these have provided adequate objective long-term follow-up or led to more widespread adoption of the results.

A report in humans of 50 patients using small intestinal submucosa without cell seeding [23] showed after a mean follow-up of 31.2 months (range 24–36 months) the excellent clinical, radiological, and cosmetic findings in 40 (80%) patients. Re-strictures developed in 1 of 10 bulbar, 5 of 31 bulbopenile, and 4 of 9 penile strictures. These all occurred in the first 6 months post-operatively. A randomised controlled trial comparing an acellular matrix with oral mucosa has also been reported [24]. Human demineralized bone matrix, obtained from cadaveric donors, was processed and prepared for use as an off-the-shelf material. 30 patients with stricture 21–59 years old (mean 36.2) were enrolled and assessed using a standard protocol. The stricture length ranged from 2 to 18 cm (mean 6.9), of which 11 patients had bulbar, 7 had pendulous and 12 had combined bulbopendulous strictures. All patients except two who were lost during follow-up were followed for 18–36 months (mean 25). In patients with a healthy urethral bed (less than two prior operations), the success rate of buccal mucosa grafts (10 of 10) was similar to the bladder matrix grafts (8 of 9) in terms of patency. In patients with an unhealthy urethral bed (more than two prior operations), only two of six patients with a bladder matrix graft were successful, whereas all five patients with a buccal mucosa graft had a patent urethra. At this time, whilst the results of these studies are of considerable interest, further information in terms of longer follow-up with objective assessment is essential before these data would lead to more widespread adoption of these techniques into routine clinical practice.

We established a programme to evaluate the potential for bio-engineering oral mucosa, because of the cases which we treat with full-length urethral strictures usually consequent upon lichen sclerosis of the urethra, where it can be difficult to be able to harvest an adequate amount of oral mucosa. In 2008, we reported the first use of tissue-engineered oral mucosa for augmentation urethroplasty in five patients. We found when used as an open graft as the first stage of a two-stage procedure of the penile urethra, that the initial results were good in all five patients with rapid vascularisation of the graft [25]. In this study, all the patients had lengthy strictures with the same aetiology, namely lichen sclerosis. Retubularisation of all five patients occurred as though native buccal mucosa had been used. However, after 8 and 9 months, respectively, two of the five patients’ grafts developed contraction and fibrosis. One graft was completely removed and one partially removed and replaced by native buccal mucosa. Histology of the two excised grafts showed pronounced epithelial hyperproliferation and fibrosis [26]. Careful follow-up and a recent 9-year follow-up in 2014 showed no stricture progression in the four patients followed up and endoscopically the mucosa was still in place and macroscopically normal [26–28]. In view of these mixed clinical results, we decided to undertake further investigation into the mechanism of contraction of tissue-engineered bladder mucosa in vitro before going forward clinically. After careful reflection, we decided in view of the ease of harvesting oral mucosa, the limited number of cases which required more than 15 cm of augmentation and the high cost of such an approach, and did not continue the project.

A new bioengineered material was reported 3 years later, in an observational study of five boys with urethral defects of length 4–6 cm with a follow-up ranging from 36 to 76 months post-operatively, using polylactide-*co*-glycolide (PLGA), seeded with autologous bladder smooth muscle and urothelial cells, and prepared as tubularised grafts. The median age was 11 years (range 10–14) at the time of surgery and median follow-up was 71 months (range 36–76 months. The median end maximum urinary flow rate was 27.1 mL/s (range 16–28), and serial radiographic and endoscopic studies showed the maintenance of wide urethral calibres without strictures. Urethral biopsies showed that the engineered grafts had developed a normal appearing architecture by 3 months after implantation [29]. These excellent data are more positive than seen in contemporary clinical experience using a tubularised autologous oral mucosal graft.

The most important factors in assessing the outcome of a new tissue engineering procedure is careful background basic research, the appropriate use of animal models [30, 31] and the comprehensive long-term follow-up of patients. A further important issue is the increased recognition in recent years that when cells, particularly stem cells, are harvested and placed in a new tissue environment then they can change their phenotype. There are clearly a number of challenges in particular when bioengineering technology is applied to organs such as the trachea. After initial optimism [32], a more measured and reflective approach has been adopted consequent upon complications seen with the clinical application of this technology [33, 34].

The importance of a carefully preclinical and clinical evaluation of any tissue-engineered oral mucosal product before it is introduced into practice has been emphasised [35, 36]..These authors highlighted the challenges associated with introducing a tissue-engineered product into clinical practice and reviewed what they felt to be the relevant regulatory challenges in Europe [37]. Clinical co-workers allied with this group expressed enthusiasm for this new material [38] and cited the results of this initial study in support of this view. In this initial series of patients (*n* = 21) with a median follow-up of 18 months (range 13–22), the success rate reported was 80.9%. This study represents the most important step in the clinical use of tissue-engineered material for urethral reconstruction [37]. Barbagli and Lazzeri concluded ‘that by following strict protocol criteria, it is possible to move tissue-engineering technology from the laboratory bench to the bedside’. They did, however, emphasise the importance of an appropriate subsequent study and were justified in taking a cautious approach as the subsequent larger prospective observational multicentre clinical study of 99 patients from eight centres Subsequently showed from this group [39]. They reported success with a heavy reliance on subjective measures and flow rates at 2 years in 98 patients. There was significant variation in the results between different centres, with two low-volume centres reporting success rates of 0% and 50%, respectively, at 1 year with an overall success rate of 67.3% and a 2-year success rate of approximately 60%. From a careful review of this paper, it is in my view likely if urethrography or urethroscopy had been carried out in all of the cases the success rate is likely to have been lower than this. This is clearly less than the success rates noted in a systematic review of more than 2000 anterior urethroplasty procedures described in the literature. For bulbar urethral strictures, there was no significant difference between the average success rates of the dorsal and the ventral onlay procedures, 88.4% and 88.8% at 42.2 and 34.4 months in 934 and 563 patients, respectively [40].

## Conclusion

Urethral strictures are common and management of long-segment strictures present a ongoing challenging surgical problem primarily because of stricture recurrence following urethrotomy or urethral dilatation This is an area where much more research is needed and we would conclude that it is an area of unmet clinical need where users of tissue engineering in the future need to carry out a rigorous basic science programme and need to be cautious in drawing conclusions based on initial experience and report on long-term clinical results.
