# Correction to: Tissue engineering of the urethra: where are we in 2019?

**DOI:** 10.1007/s00345-019-02929-x

**Published:** 2019-08-30

**Authors:** Christopher Chapple

**Affiliations:** Sheffield Teaching Hospitals, Sheffield, UK

## Correction to: World Journal of Urology 10.1007/s00345-019-02826-3

It has been brought to our attention that the first sentence of the Conclusion in the original publication [1] is incorrect. We have removed this sentence and the correct version can be found in this Erratum.

Conclusion:

Urethral strictures are common and management of long-segment strictures present a ongoing challenging surgical problem primarily because of stricture recurrence following urethrotomy or urethral dilatation This is an area where much more research is needed and we would conclude that it is an area of unmet clinical need where users of tissue engineering in the future need to carry out a rigorous basic science programme and need to be cautious in drawing conclusions based on initial experience and report on long-term clinical results.

Reference:

1. Chapple C (2019) Tissue engineering of the urethra: where are we in 2019? World J Urol. 10.1007/s00345-019-02826-3.

The original article has been corrected.

